# Interaction Effect between Elevated CO_2_ and Fertilization on Biomass, Gas Exchange and C/N Ratio of European Beech (*Fagus sylvatica* L.)

**DOI:** 10.3390/plants5030038

**Published:** 2016-09-07

**Authors:** Neda Lotfiomran, Michael Köhl, Jörg Fromm

**Affiliations:** 1 Institute for World Forestry, University of Hamburg, Leuschnerstrasse 91, 21031 Hamburg, Germany; Michael.koehl@uni-hamburg.de; 2 Institute for Wood Biology, University of Hamburg, Leuschnerstrasse 91d, 21031 Hamburg, Germany

**Keywords:** climate change, elevated CO_2_, fertilization, interaction effect, European beech, biomass, gas exchange, C/N ratio

## Abstract

The effects of elevated CO_2_ and interaction effects between elevated CO_2_ and nutrient supplies on growth and the C/N ratio of European beech (*Fagus sylvatica* L.) saplings were studied. One-year-old beech saplings were grown in a greenhouse at ambient (385 ppm) and elevated CO_2_ (770 ppm/950 ppm), with or without fertilization for two growing seasons. In this study, emphasis is placed on the combined fertilization including phosphorus, potassium and nitrogen with two level of elevated CO_2_. The fertilized plants grown under elevated CO_2_ had the highest net leaf photosynthesis rate (Ac). The saplings grown under elevated CO_2_ had a significantly lower stomatal conductance (gs) than saplings grown under ambient air. No interaction effect was found between elevated CO_2_ and fertilization on Ac. A interaction effect between CO_2_ and fertilization, as well as between date and fertilization and between date and CO_2_ was detected on gs. Leaf chlorophyll content index (CCI) and leaf nitrogen content were strongly positively correlated to each other and both of them decreased under elevated CO_2_. At the end of both growing seasons, stem dry weight was greater under elevated CO_2_ and root dry weight was not affected by different treatments. No interaction effect was detected between elevated CO_2_ and nutrient supplies on the dry weight of different plant tissues (stems and roots). However, elevated CO_2_ caused a significant decrease in the nitrogen content of plant tissues. Nitrogen reduction in the leaves under elevated CO_2_ was about 10% and distinctly higher than in the stem and root. The interaction effect of elevated CO_2_ and fertilization on C/N ratio in plants tissues was significant. The results led to the conclusion that photosynthesis and the C/N ratio increased while stomatal conductance and leaf nitrogen content decreased under elevated CO_2_ and nutrient-limited conditions. In general, under nutrient-limited conditions, the plant responses to elevated CO_2_ were decreased.

## 1. Introduction

Carbon dioxide is a natural component of the atmosphere and an essential factor for the growth of trees since nearly half of their constituents are composed of carbon. The CO_2_ content in the ambient atmosphere has not been constant through time. Since around 1850, it has continuously risen from about 270 ppm to around 400 ppm at present and will continue to do so in future [1]. Carbon dioxide emissions are the most important cause of global warming. As a sink for CO_2_, forests play an important role in the discussion about global warming [2]. The recent rise of the CO_2_ content in the ambient air and its implications for the biosphere, including global warming, have been a focus of politics for more than two decades, and numerous studies have analyzed the effects of elevated CO_2_ on plants [3,4,5,6,7,8,9]. A large body of information has also been produced in response to the effect of elevated CO_2_ on trees [6,8,9,10,11,12,13,14]. Despite this vast number of CO_2_ related papers published to date, however, more research on trees and shrubs under elevated CO_2_ is still needed. Nevertheless, a considerable but heterogeneous variety of experimental results and a wide diversity of interpretations have become accessible.

Photosynthesis is directly affected by a varying CO_2_ content in the ambient air and is therefore one of the major physiological processes being monitored during all kind of experiments. Numerous short-term studies have showed an increase in the photosynthesis rate caused by an elevated CO_2_ concentration [3,5,6,10,11,15,16,17]. However, the photosynthetic behaviour of trees under long-term exposure to elevated CO_2_ is less clear [12]. Although it is generally agreed that short-term growth under elevated CO_2_ causes a stimulation of the photosynthesis rate in trees, it has frequently been reported that this enhancement may decline or even disappear with time [18,19]. Furthermore, in several studies a “down regulation” of the photosynthesis rate was shown in plants grown under elevated CO_2_ over longer periods (weeks or months) [9,11,13,20].

The production of biomass is one of many paths along which carbon is metabolized [9]. Several measurements on seedlings and young trees indicate that total growth increases with an increased carbon uptake [6,8,11,21,22,23]. There is also strong evidence that plant biomass production under elevated CO_2_ is mostly larger than in ambient air, although a number of studies showed no significant increase, or even a decrease, of biomass production under elevated CO_2_ [7,24,25]. In those studies that do show an increase, the magnitude of biomass enhancement varies widely between observations. The enhancing effect of elevated CO_2_ on biomass production can decline or totally disappear with time [11,18,26,27,28]. Growth stimulation, as well as other tree responses to elevated CO_2_ depends on various factors such as the developmental stage of the plants [29,30], genetic factors, environmental conditions [6,31] and water and nutrient availability [6,8,32,33,34]. Therefore several investigators have focused on the interaction of elevated CO_2_ with, for example, drought [35], temperature [36], and other greenhouse gases such as O_3_, SO_2_ and NO_3_ [37,38,39] as well as nutrient availability [40,41]. The plant responses to elevated CO_2_, decreased under nutrient limited conditions but it is reported that this tendency was weak and mostly readily apparent in plants which were grown with severe nutrient limitation [42]. However, it is difficult to generalize the plant responses under elevated CO_2_ to nutrient limitations.

Nitrogen is part of all living cells and of all proteins, enzymes and metabolic processes involved in the synthesis and transfer of energy. Trees require a considerable amount of N for their growth, especially when growing faster under elevated CO_2_ [33]. One of the most common observations is a lower N concentration in plant components grown under elevated CO_2_ than in ambient air [20,27,28,43,44,45].

One of main species in deciduous forests in Europe is the European beech (*Fagus sylvatica* L.). The distribution of the European beech is wide, although concentrated in west and central Europe. With a 10.3 m^3^/ha/year growth rate, after Spruce (*Picea abies*), beech is the second fastest growing tree species in European forests [46]. Due to this importance, it is necessary to understand how European beech responses to global warming, caused mainly by enrichment of CO_2_ concentration in the atmosphere. Elevated CO_2_ caused changes in the wood structure and anatomical characteristics of European beech [36,47]. Increases in both the photosynthesis rate and biomass of European beech under elevated CO_2_ have been reported in previous studies [18,48,49]. On the other hand, it is now widely accepted that the initial stimulation of photosynthesis under elevated CO_2_ is often followed by a decline in photosynthesis, usually accompanied by a decrease in nutrient content, especially nitrogen in plant tissues.

The response of European beech to high CO_2_ can be influenced by an interaction effect between elevated CO_2_ and other factors such as temperature, drought and other greenhouse gases [36,37,38,50]. Although numerous studies dealt with responses of beech tree growth under elevated CO_2_, combined experiments with CO_2_ and nutrient application are rare and the fertilizer treatments are mostly based on nitrogen [50]. In the present study, one-year-old potted beech saplings with and without fertilization were grown under different levels of elevated CO_2_ (770/950 ppm) in a greenhouse for two growing seasons. The effects of elevated CO_2_ and fertilization on the photosynthesis rate (Ac), stomatal conductance (gs) and leaf chlorophyll content were investigated. The variation of biomass allocation and C/N ratio between different treatments were also studied. In this study, emphasis is placed on the combined fertilization including phosphorus, potassium and nitrogen with elevated CO_2_. The main objective of the present study was to investigate how different levels of nutrient supplies interact with the effects of elevated CO_2_ on European beech. 

## 2. Results

### 2.1. Variation of Height Growth and Dry Weight of Leaves, Stem, Root and Total Biomass between the Treatments

**Figure 1:**
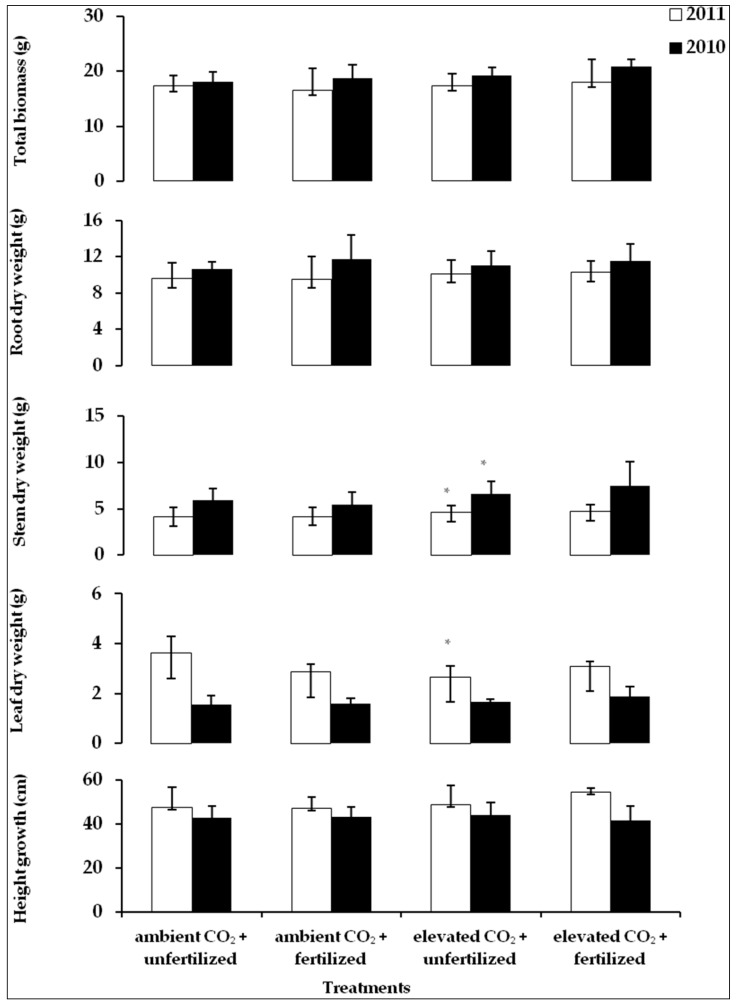
Effect of elevated CO_2_ (770 ppm in 2010 (□), 950 ppm in 2011 (■)) and fertilization on mean values (± st.dev.) of height growth, leaf dry mass, stem dry mass, root dry mass and the total biomass at the end of both growing seasons, (*n* = 10). An asterisk indicates significant differences between treatments (*p* ≤ 0.05).

Regarding height growth, no significant differences were observed between different treatments in both years (Figure 1), as supported by statistical analysis (Table 1). The variation of stem dry weight between treatments showed a similar pattern in both growing seasons (Figure 1). Plants grown under elevated CO_2_ conditions produced significantly heavier stems (Figure 1), but the differences between fertilized and unfertilized plants grown under different CO_2_ levels (elevated-ambient) were not significant (Table 1). No interaction effect between elevated CO_2_ and fertilization on stem dry weight was found. In 2010, no significant changes in the dry weight of leaves were observed between different treatments. In 2011, however, the dry weight of the leaves varied significantly between treatments (Figure 1, Table 1). Unfertilized plants under ambient air (control) had the greatest leaf weight; in contrast, unfertilized saplings under elevated CO_2_ had the lowest value. The interaction between CO_2_ and fertilization was significant. Hence, fertilization alone had no effect on dry weight but under elevated CO_2_, the difference between the dry weight of the leaves of unfertilized and fertilized plants was significant (Table 1). In both years, on average, 50% of the total biomass consisted of root dry mass and differences in root dry mass as well as total biomass of treatments were unaffected by either CO_2_ concentration of fertilization (Table 1). 

### 2.2. Seasonal Variations in Photosynthesis Rate and Stomatal Conductance 

**Figure 2:**
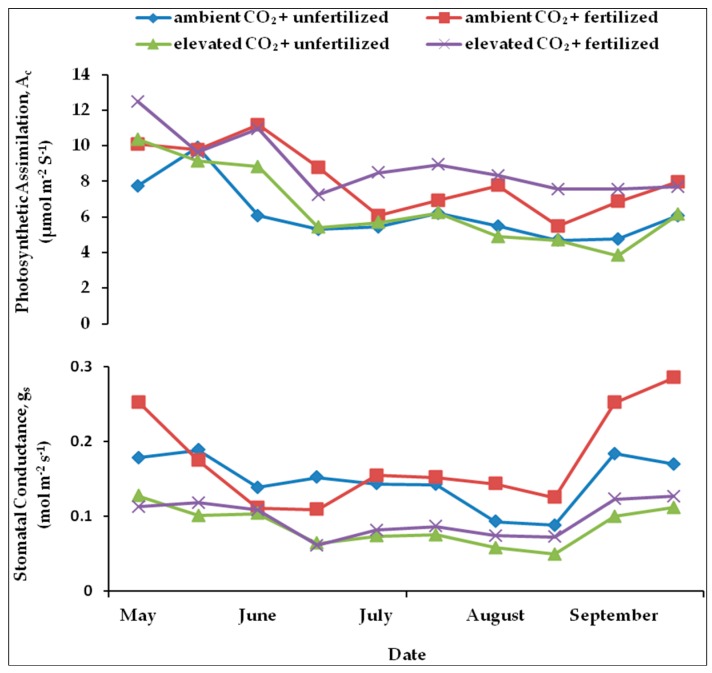
The ratio of net photosynthetic rate (Ac) (above) and stomatal conductance (gs) (below) under ambient CO_2_ (385 ppm) or elevated CO_2_ (950 ppm) with different levels of nutrient availability during the second growing season (May-September 2011). Values are means of 10 replicates for each treatment. (◊; ambient CO_2_ + unfertilized), (■: ambient CO_2_ + fertilized), (Δ; elevated CO_2_ + unfertilized) and (x; elevated CO_2_ + fertilized).

The seasonal tendencies in photosynthesis rate (Ac) and stomatal conductance (g_s_) for the different treatments in 2011 are summarized in Figure 2. The maximum mean value of Ac occurred with fertilized saplings grown under elevated CO_2_ (950 ppm). The CO_2_ uptake rate of fertilized plants grown under different CO_2_ concentrations was higher than in unfertilized plants during the whole growing season (Figure 2). Repeated measures ANOVA showed a significant effect of elevated CO_2_ and fertilization as well as measurement date on Ac. Both elevated CO_2_ and fertilization caused an increase in Ac and during the growing season (May–September), the rate of net photosynthesis decreased significantly (Table 2). Significant interactions also occurred between fertilization and elevated CO_2_ but no interaction effects were detected between date and CO_2_ and between date and fertilization (Table 2).

Statistical analysis showed gs to be significantly affected by CO_2_, fertilization and date of samplings (Table 2). Saplings under ambient CO_2_ had significantly higher gs during the whole growing season. At the end of the growing season, the respective highest and lowest value of gs was observed in fertilized saplings under ambient CO_2_ and unfertilized plants under elevated CO_2_, (Figure 2). During the growing season, the increasing trend in gs was significant (Table 2). Elevated CO_2_ significantly decreased gs; in contrast, fertilization caused a significant increase in this parameter (Table 2). There was also an interaction effect between CO_2_ and fertilization, as well as between date and fertilization and between date and CO_2_ on gs. 

### 2.3. Chlorophyll Concentration Index (CCI) in Leaves

**Figure 3:**
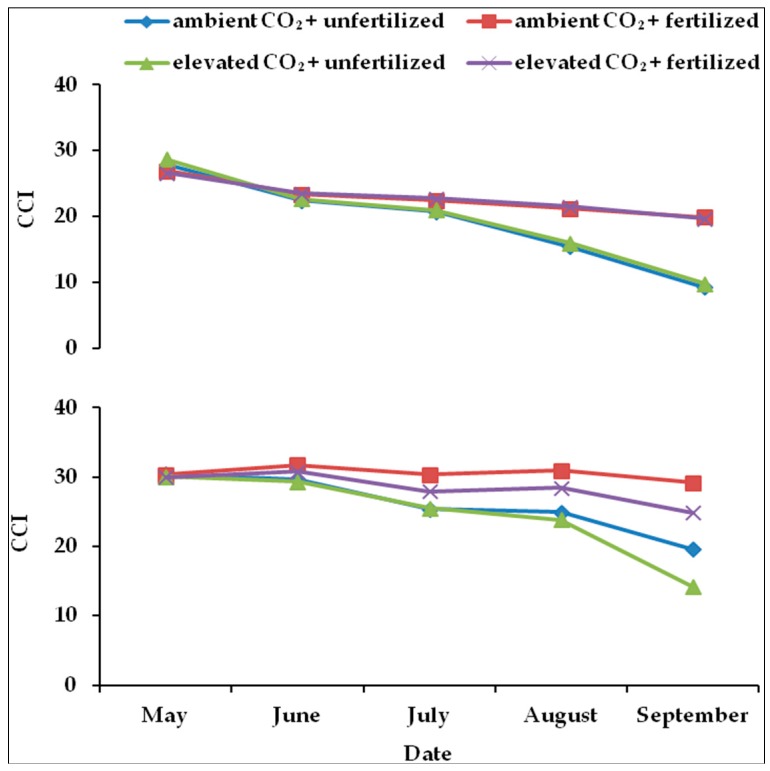
The variation of the mean chlorophyll concentration index (CCI) under ambient CO_2_ (385 ppm) or elevated CO_2_ (770 ppm during the first growing season (above) and 950 ppm during the second growing season (below)) with different levels of nutrient availability. Values are means of 10 replicates for each treatment. (◊; ambient CO_2_ + unfertilized), (■: ambient CO_2_ + fertilized), (Δ; elevated CO_2_ + unfertilized) and (x; elevated CO_2_ + fertilized).

During both growing seasons, the rate of decline in CCI of unfertilized plants was significantly faster than that in fertilized plants, except in May. In addition, no interaction effect between fertilization and CO_2_ concentration could be detected (Table 3, Figure 3). Elevated CO_2_ reduced CCI significantly in 2011 (Table 3). 

### 2.4. Correlation between Nitrogen Content and CCI at the end of Growing Seasons

**Figure 4:**
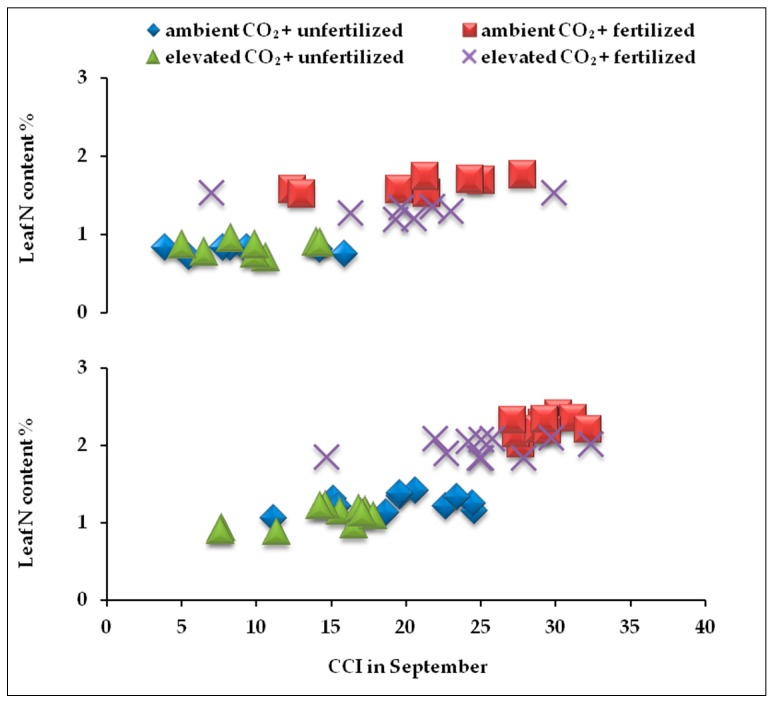
Correlation between CCI and leaf nitrogen content in the leaf at the end of the first (above) and the second (below) growing season.

In both years, at the end of the growing season (September), the highest values of leaf nitrogen content were measured in fertilized plants grown under ambient air (Figure 4). Both parameters, CCI and nitrogen, were strongly positively correlated with each other (Table 3).

### 2.5. C/N Ratio in Plants Tissues

**Figure 5:**
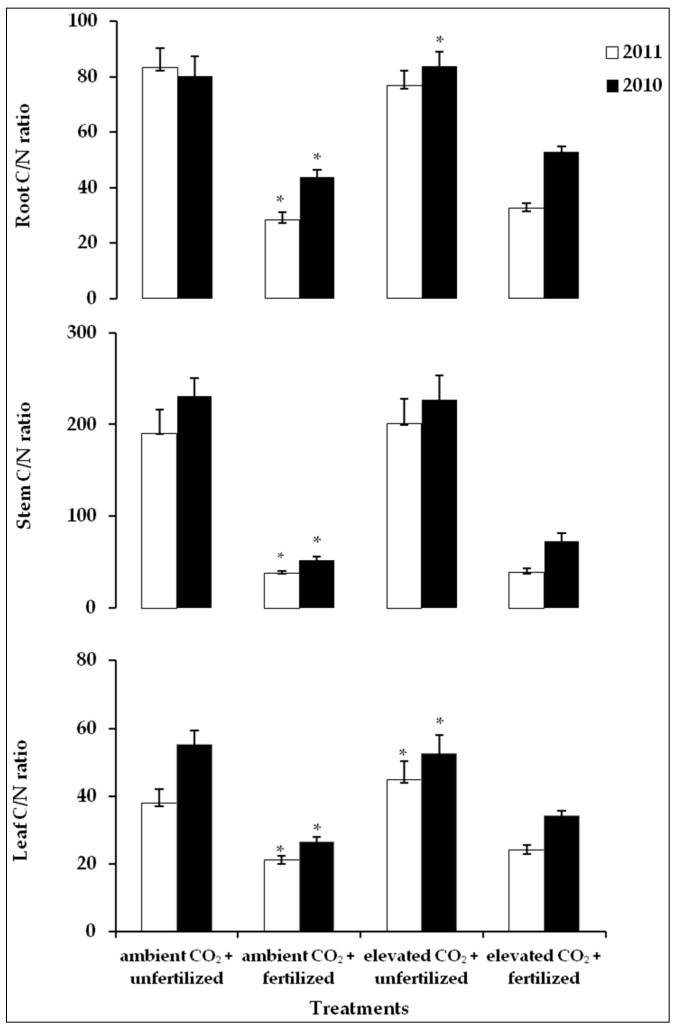
Effect of elevated CO_2_ (770 ppm in 2010 (□), 950 ppm in 2011 (■)) and fertilization on mean values of the C/N ratio in leaf, stem and root, (*n* = 10). An asterisk indicates significant differences between treatments (*p* ≤ 0.05).

The C/N ratio in the leaves varied significantly from 26.6%–55.3% in 2010 and from 21.0%–44.9% in 2011 (Figure 5). In both growing seasons, the lowest value was measured in fertilized plants growing under ambient air (Figure 5). Fertilization caused a significant decrease in the C/N ratio because of rising N content. Elevated CO_2_ increased this ratio in both years (Figure 5), and in both years, the interaction effect between elevated CO_2_ and fertilization was significant. Regarding the stems, the C/N ratio varied substantially among treatments in both experimental years (Figure 5). The differences in the mean values between treatments showed the same trend in both growing seasons (Figure 5). Fertilized plants grown under either of the CO_2_ concentrations (ambient air/elevated) had a significantly lower C/N ratio, but the effects of the CO_2_ concentration on the C/N ratio were statistically insignificant (Table 4).

The mean values of the C/N ratio in the roots ranged from 43.8%–83.7% in 2010 and from 28.3%–83.3% in 2011 (Figure 5). In both growing seasons, the lowest ratio was measured in fertilized plants grown under ambient air. Similar to the leaf and stem, fertilization resulted in a decrease in the C/N ratio in roots (Figure 5). A significant effect of elevated CO_2_ concentration on the C/N ratio was only detected in 2010 (Table 4).

## 3. Discussion

### 3.1. Phenological Responses to Elevated CO_2_

The study showed that the height of the saplings varied between the treatments but was not statistically significant. This may be due to the large spread of data acquired per treatment. Although Overdieck et al. [36] mentioned a positive influence of elevated CO_2_ on height growth of beech, generalizing from these earlier results for beech appears to be difficult because of the differing experimental conditions and genotypes of the study material. The increase in the stem weight of beech saplings grown under elevated CO_2_ was the most obvious and significant response in this study (Figure 1). In both study years, the stems were heavier when grown under elevated CO_2_ than under ambient air. In general, it appears that beech produces more biomass under elevated CO_2_ [36,49]. In this study, however, the total biomass was not affected by elevated CO_2_. This might be due to the fact that half of the biomass consisted of roots, which remained unaffected by the differing growth conditions [50]. No interaction effect was found between elevated CO_2_ and fertilization on the dry weight of saplings during either of the growing seasons. 

### 3.2. Physiological Responses to Elevated CO_2_

Photosynthesis throughout the growing season was significantly higher in fertilized saplings under both ambient and elevated CO_2_ (Figure 2). The positive effects of elevated CO_2_ on the rate of photosynthesis of beech have been previously reported [36,51] but a contrary effect or no significant effect at all [49] has also sometimes been observed. At the beginning of the growing season, stimulation of the photosynthesis rate was observed in all saplings (fertilized and unfertilized) under elevated CO_2_ but this increase disappeared very soon in unfertilized plants. This reduction was in part due to a decrease in N content and could also be a consequence of the potential sink limitations imposed by pot size. Further, with respect to fertilization, a positive effect under elevated CO_2_ has been repeatedly described [52].

Both leaf nitrogen content and the chlorophyll content index in plants under elevated CO_2_ were significantly less than in plants grown under ambient air. The decrease in leaf chlorophyll content in beech under elevated CO_2_ was reported [38,53], which may be a consequence of leaf nitrogen dilution [8]. Part of the decrease in the photosynthesis rate could be caused by the loss of chlorophyll in plants which were grown under elevated CO_2_ during the growing season.

A clear effect of the different treatments on the stomatal conductance of the saplings could be seen in the second year, when elevated CO_2_ resulted in a significant reduced stomatal conductance, [51]. In the short term, greater depolarization under elevated CO_2_ will result in reduced stomatal aperture [17]. In the long term, changes in stomatal density or stomatal index as well as stomatal aperture can be caused by the reduction of stomatal conductance under elevated CO_2_ [17]. On the other hand, no significant reduction of the stomatal conductance of beech under elevated CO_2_ was observed by Heath and Kerstiens [50]. Interestingly, the rate of stomatal conductance was significantly higher in fertilized saplings compared to unfertilized ones (Figure 2; Table 2). 

### 3.3. Biochemical Responses to Elevated CO_2_

The C/N ratio can be increased, decreased or left unaffected by elevated CO_2_, but, on average, increases by about 15% when the CO_2_ concentration was doubled [54]. As we know, elevated CO_2_ causes an increase in photosynthetic activity [6,11,16]; therefore, an elevated C content was observed in all components of the saplings grown under elevated CO_2_. In fertilized saplings, a higher N content was detected in plant organs, but the C content remained unaffected by fertilization. In addition, a reduction in the nitrogen content in plants grown under elevated CO_2_ has been observed very often in the past [23,34,43,44,55]. What is the reason for such a reduction? The cause of this reduction is not clear, and the physiological mechanisms responsible for this observation have not been definitely established, although a considerable number of hypotheses have been advanced to account for it [45]. One contributing factor is dilution of N by increased photosynthetic assimilation of C. In addition, studies show strong evidence for a general decrease in the uptake of N per unit mass or length of roots under elevated CO_2_. This decreased uptake appears to be the result of both a decreased N demand by shoots and of a decreased ability of the soil-root system to supply the plant with N. The best-supported mechanism for a decreased N supply is a decrease in the transpiration-driven flow of N in the soil due to a decreased stomatal conductance at elevated CO_2_. Another hypothesis indicates that altered root-system architecture may also play a role [45]. There is also limited evidence suggesting that under elevated CO_2_, plants may exhibit increased rates of N loss through volatilization and/or root exudation, further contributing to lowering the N concentrations [45]. In this study, the N reduction under elevated CO_2_ amounted to 10% in the leaves but distinctly less in the stems and roots. Similar observations were reported by Cotrufo et al. [43]. In our saplings, the C/N ratio was higher when grown under elevated CO_2_ (Figure 5). Certainly, one contributing factor is the dilution of N by increased CO_2_ uptake. Reduction in N uptake can also be due to decreased transpiration and stomatal conductance [56,57,58]. Such a decrease of stomatal conductance under elevated CO_2_ was detected in the saplings in the present experiment. Although some evidence suggests that altered root-system architecture may also play a role [41], in this study, no significant differences in root architecture were found between treatments. Due to an increase in N by fertilization, the resulting C/N ratio was lower in fertilized saplings grown under different CO_2_ concentration.

## 4. Materials and Methods 

The seedlings of European beech (*Fagus sylvatica* L.), were collected from a tree nursery near Hamburg and grown in the institute’s greenhouse in Hamburg, located at 53°30′ N and 10°12′ E at an elevation of 25 m a.s.l., during two growth seasons (May to September 2010–2011). In spring, the one-year-old saplings were planted in 1 litre tops filled with 50% sand and 50% of a standard commercial substrate (TKS1), including 140 mg/L N, 80 mg/L P_2_O and 190 mg/L K_2_O; pH 5.6. 

### 4.1. Growth Conditions and Experimental Design

The saplings were randomly assigned to two chambers with different CO_2_ concentrations in the greenhouse. In one of them, the CO_2_ concentration was the same as in ambient air (on average 385 ppm). In the other one, the CO_2_ concentration was raised to either 770 ppm in the first growing season (2010) or 950 ppm in in the second growing season (2011). In the second year, the CO_2_ concentration increased by 200% to investigate if this increase reinforced the responses of the saplings to elevated CO_2_. In each chamber, the saplings were divided into two groups with different nutrient supply rates (fertilized and unfertilized). For the fertilized group, 0.2% NPK liquid fertilizer (Wuxal Top N) was applied once per week during the growing season.

From each of four treatment combinations of CO_2_ concentration and nutrient supply (ambient CO_2_ + unfertilized (control), ambient CO_2_ + fertilized, elevated CO_2_ + unfertilized, elevated CO_2_ + fertilized), 10 plants were analyzed. All plants were irrigated weekly with 80 mL of tap water. 

During the growing season, the temperature in the greenhouse was kept constant at about 20 °C. The length of the photoperiod was the same as in the Hamburg area and the relative air humidity (RH) was about 70%. During the experiments, the CO_2_ level, temperature, photoperiod and air humidity were monitored by the Computer Climate model CC 600 (RAM Co. Measurement and Control, Germany) every 12 min. Leaf gas exchange parameters were measured many times during the night (from 21:00) and it was found that the photosynthesis activities were about zero. The CO_2_ levels (ambient air, 770 and 950 ppm) were maintained between 7:00 and 21:00 each day, which was matched to the photoperiod. Moreover, all plants were inspected once a week during the growing season.

### 4.2. Seasonal Gas Exchange Measurements

During the second growing season (2011), regular measurements of the net photosynthesis rate (A_C_) and stomatal conductance (gs) were made using a portable Infra-Red Analyzer (IRGA; LI-6400, Li-Cor., Lincoln, NE. USA) on one leaf of each sapling. It was considered that the chosen leaves for gas exchange measurements were of similar age. The rate of air flow through the system was set to 600 µmol s^−1^ and the light intensity, provided by a red-blue light source was set to 800 µmol m^−2^ s^−1^. The CO_2_ concentration in the chambers was controlled regularly and measurements were performed at the CO_2_ concentrations under which plants were grown.

The air humidity was regulated by adjusting the air flow through a desiccant tube. The chlorophyll content index (CCI) of the leaves of all saplings was non-destructively determined by a chlorophyll meter (SPAD-502 plus Konica Minolta) once a week on 15–20 leaves per sapling during both growing seasons. The values measured correspond to the percentage of chlorophyll in the leaves. They were calculated from the amount of light, transmitted through a leaf at two wavelengths for which the absorbance of chlorophyll is different. 

### 4.3. Growth Measurements

At the end of the each growing season, the tree height was measured and then all trees were harvested and separated into leaves, stems and roots. After the samples were dried at 70 °C for one week, the dry weight of these three fractions was determined and the sum was calculated as a total biomass.

### 4.4. C/N Ratio

After having determined the dry weight of the various organs, the leaves, stems without bark and roots were ground to a powder using a mill. Then, 5–10 mg of the powder was filled into tin capsules and the total carbon and nitrogen contents of each sample were measured using an element analyzer instrument (Vario EL cube; Hanau, Germany). During analysis, the temperature in the oxidation oven was 1050 °C, while in the thermal conductivity detector and chromatographic column it was 115 °C. The carrier gas pressure was 80 kPa and the flow rate 125 mL/min. The oxygen addition was 20 mL and the oxygen pressure was 50 kPa.

### 4.5. Statistical Analysis

The main effects of elevated CO_2_, fertilization and their interaction were evaluated with a general linear model. To evaluate the gas exchange data during the growing season, repeated measures multivariate ANOVA was applied. The Pearson’s correlation test was used to check the correlation between the chlorophyll concentration index and leaf N content at the end of the growing season. The normality of distribution and equality was checked using the Kolmogorov-Smirnov test. The data for each experimental year were analyzed separately and independently from each other. The statistical significance was set at *p* ≤ 0.05 for all tests. Standard deviation (SD) was used to show the distribution of the data around the mean. All statistical analyses were performed using SPSS 18 (SPSS Inc., Chicago, IL, USA) and a Complete Randomized Design (CRD) was considered for the greenhouse experiments.

## 5. Conclusions 

To find out how trees respond to elevated CO_2_ in combination with nutrient availability, we investigated the interaction between elevated CO_2_ and different levels of nutrient supply on European beech saplings. The interaction between elevated CO_2_ and fertilization on photosynthetic activities (Ac) of beech was not statistically significant. Generally enhanced tree growth by elevated CO_2_ was greater than by fertilization. Negative interaction effects between CO_2_ and fertilization were found in the C/N ratio of leaves and roots. The reduction of N content under elevated CO_2_ in plant organs was observed, especially in leaves [43]. Such a decrease in N content obviously affects the nutrient composition of plant material used by herbivores. Therefore, elevated CO_2_ has complex effects on the whole ecosystem level. Future studies will also focus on changes in other macronutrients, such as potassium, phosphorus and calcium, under increasing CO_2_ levels.

## Figures and Tables

**Table 1:** Statistical strength (F and *p*-values) of the effects of fertilization and CO_2_ concentrations on height, leaf dry mass, stem dry mass and root dry mass between treatments; * *p* ≤ 0.05; ** *p* ≤ 0.01; *** *p* ≤ 0.001.

		Height		Leaf		Stem		Root		Total biomass	
		F	*p*-value	F	*p*-value	F	*p*-value	F	*p*-value	F	*p*-value
**CO_2_ concentration**	2010	2.88	0.10	3.27	0.08	5.10 *	0.03	0.02	0.88	2.18	0.15
	2011	0.00	0.98	6.55 **	0.01	4.36 *	0.04	2.32	0.14	1.76	0.19
**Fertilization**	2010	1.16	0.29	1.35	0.25	0.15	0.70	1.53	0.22	1.05	0.31
	2011	0.69	0.41	1.70	2.00	0.16	0.90	0.42	0.52	0.02	0.88
**CO_2_ × Fertilization**	2010	1.65	0.21	0.87	0.36	1.30	0.26	0.14	0.70	0.25	0.62
	2011	1.13	0.30	15.2 ***	0.00	0.00	0.95	0.58	0.45	2.18	0.15

**Table 2:** Results (MS; Mean Square, F statistics and *p*-values) from repeated sample measure ANOVA for net photosynthetic rate (Ac) and stomatal conductance (gs) of saplings during the growing season. * *p* ≤ 0.05; ** *p* ≤ 0.01; *** *p* ≤ 0.001.

Source	Ac			gs		
	MS	F	*p*-value	MS	F	*p*-value
**CO_2_ concentration**	28.02	5.54 *	0.021	0.490	397.5 ***	0.000
**Fertilization**	439.24	86.83 ***	0.000	0.036	29.6 ***	0.000
**CO_2_ × Fertilization**	3.22	0.636	0.428	0.008	6.86 *	0.011
**Date**	620.55	165.25 ***	0.000	0.097	61.394 ***	0.000
**Date × CO_2_**	6.49	1.73	0.193	0.014	8.592 ***	0.000
**Date × Fertilization**	5.43	1.44	0.233	0.015	9.228 ***	0.000

**Table 3:** Statistical strength (*p*-values) of the effects of fertilization and CO_2_ concentrations on the CCI between the treatments and correlations (Pearson Correlation Coefficient) between the CCI and the nitrogen content in leaves; * *p* ≤ 0.05; ** *p* ≤ 0.01; *** *p* ≤ 0.001.

		CO_2_ concentration	Fertilization	CO_2_ × Fertilization	Leaf nitrogen content
**CCI**		*p*-value	*p*-value	*p*-value	*values of Pearson correlation*
	2010	0.95	0.000 ***	0.83	0.74 **
	2011	0.000 ***	0.000 ***	0.67	0.85 **

**Table 4:** Statistical strength (F and *p*-values) of the effects of fertilization and CO_2_ concentrations on the C/N ratio of leaf, stem and root between the treatments; * *p* ≤ 0.05; ** *p* ≤ 0.01; *** *p* ≤ 0.001.

Treatments		C/N ratio					
		Leaf		Stem		Root	
		F	*p*-value	F	*p*-value	F	*p*-value
**CO_2_ concentration**	2010	10.26 **	0.003	3.02	0.09	6.83 *	0.012
	2011	38.67 ***	0.000	1.84	0,18	1.23	0.27
**Fertilization**	2010	588.55 ***	0.000	1122 ***	0.000	201.5 ***	0.000
	2011	574.55 ***	0.000	1378 ***	0.000	2155 ***	0.000
**CO_2_ × Fertilization**	2010	20.49 ***	0.000	3.90	0.12	1.43	0.24
	2011	6.10 *	0.016	1.33	0.25	24.5 ***	0.000
